# Comparison of Structural Parsers and Neural Language Models as Surprisal Estimators

**DOI:** 10.3389/frai.2022.777963

**Published:** 2022-03-03

**Authors:** Byung-Doh Oh, Christian Clark, William Schuler

**Affiliations:** Department of Linguistics, The Ohio State University, Columbus, OH, United States

**Keywords:** sentence processing, incremental parsers, language models, surprisal theory, self-paced reading, eye-tracking, fMRI

## Abstract

Expectation-based theories of sentence processing posit that processing difficulty is determined by predictability in context. While predictability quantified *via* surprisal has gained empirical support, this representation-agnostic measure leaves open the question of how to best approximate the human comprehender's latent probability model. This article first describes an incremental left-corner parser that incorporates information about common linguistic abstractions such as syntactic categories, predicate-argument structure, and morphological rules as a computational-level model of sentence processing. The article then evaluates a variety of structural parsers and deep neural language models as cognitive models of sentence processing by comparing the predictive power of their surprisal estimates on self-paced reading, eye-tracking, and fMRI data collected during real-time language processing. The results show that surprisal estimates from the proposed left-corner processing model deliver comparable and often superior fits to self-paced reading and eye-tracking data when compared to those from neural language models trained on much more data. This may suggest that the strong linguistic generalizations made by the proposed processing model may help predict humanlike processing costs that manifest in latency-based measures, even when the amount of training data is limited. Additionally, experiments using Transformer-based language models sharing the same primary architecture and training data show a surprising negative correlation between parameter count and fit to self-paced reading and eye-tracking data. These findings suggest that large-scale neural language models are making weaker generalizations based on patterns of lexical items rather than stronger, more humanlike generalizations based on linguistic structure.

## 1. Introduction

Much work in sentence processing has been dedicated to studying differential patterns of processing difficulty in order to shed light on the latent mechanism underlying incremental processing. Within this line of work, expectation-based theories of sentence processing (Hale, 2001; Levy, 2008) have posited that processing difficulty is mainly driven by predictability in context, or how predictable upcoming linguistic material is given its context. In support of this position, predictability quantified through information-theoretic surprisal (Shannon, 1948) has been shown to strongly correlate with behavioral and neural measures of processing difficulty (Hale, 2001; Demberg and Keller, 2008; Levy, 2008; Roark et al., 2009; Smith and Levy, 2013; van Schijndel and Schuler, 2015; Hale et al., 2018; Shain, 2019; Shain et al., 2020, inter alia). However, as surprisal can be calculated from any probability distribution defined over words and therefore makes minimal assumptions about linguistic representations that are built during sentence processing, this leaves open the question of how to best estimate the human language comprehender's latent probability model.

In previous studies, two categories of natural language processing (NLP) systems have been evaluated as surprisal-based cognitive models of sentence processing. The first are language models (LMs), which directly define and estimate a conditional probability distribution of a word given its context. Surprisal estimates from several well-established types of LMs, including *n*-gram models, Simple Recurrent Networks (SRN; Elman, 1991), and Long Short-Term Memory networks (LSTM; Hochreiter and Schmidhuber, 1997), have been compared against behavioral measures of processing difficulty (e.g., Smith and Levy, 2013; Goodkind and Bicknell, 2018; Aurnhammer and Frank, 2019). More recently, Transformer-based (Vaswani et al., 2017) models trained on massive amounts of data have dominated many NLP tasks (Devlin et al., 2018; Liu et al., 2019; Brown et al., 2020), causing a surge of interest in evaluating whether these models acquire a humanlike understanding of language. As such, both large pretrained and smaller “trained-from-scratch” Transformer-based LMs have been evaluated as models of processing difficulty (Hao et al., 2020; Wilcox et al., 2020; Merkx and Frank, 2021).

The second category of NLP systems are incremental parsers, which make explicit decisions and maintain multiple hypotheses about the linguistic structure associated with the sentence. Surprisal can be calculated from prefix probabilities of the word sequences at consecutive time steps by marginalizing over these hypotheses. In this case, surprisal can be derived from the Kullback–Leibler divergence between the two probability distributions over hypotheses and can be interpreted as the amount of “cognitive effort” taken to readjust the hypotheses after observing a word (Levy, 2008). Examples of incremental parsers that have been applied as models of sentence processing include Earley parsers (Hale, 2001), top-down parsers (Roark et al., 2009), Recurrent Neural Network Grammars (Dyer et al., 2016; Hale et al., 2018), and left-corner parsers (van Schijndel et al., 2013; Jin and Schuler, 2020).

This article aims to contribute to this line of research by first presenting an incremental left-corner parser that incorporates information about common linguistic abstractions as a computational-level (Marr, 1982) model of sentence processing. This parser makes explicit predictions about syntactic tree nodes with rich category labels from a generalized categorial grammar (Ajdukiewicz, 1935; Bar-Hillel, 1953; Bach, 1981; Nguyen et al., 2012) as well as their associated predicate-argument structure. Additionally, this parser includes a character-based word generation model which defines the process of generating a word from an underlying lemma and a morphological rule, allowing the processing model to capture the predictability of given word forms in a fine-grained manner.

Subsequently, we evaluate this parser as well as a range of other LMs and incremental parsers from previous literature on their ability to predict measures of processing difficulty from human subjects, including self-paced reading times, eye-gaze durations, and blood oxygenation level-dependent (BOLD) signals collected through fMRI. Our experiments yield two main findings. First, we find that our structural processing model achieves a strong fit to latency-based measures (i.e., self-paced reading times and eye-gaze durations) that is comparable and in many cases superior to large-scale LMs, despite the fact that the LMs are trained on much more data and show lower perplexities on test data. Second, experiments using Transformer-based GPT-2 models (Radford et al., 2019) of varying capacities that share the same primary architecture and training data show a surprising negative correlation between parameter count and fit to self-paced reading and eye-tracking data. In other words, Transformer models with fewer parameters were able to make better predictions when the training data was held constant.

These results suggest that the strong linguistic generalizations made by incremental parsers may be helpful for predicting humanlike processing costs that manifest in latency-based measures, even when the amount of training data is limited. In addition, they add a new nuance to the relationship between language model perplexity and psychometric predictive power noted in recent psycholinguistic studies. While the comparison of neural LMs and incremental parsers mostly supports the linear relationship first reported by Goodkind and Bicknell (2018), our structural parser and the different variants of GPT-2 models provide counterexamples to this trend. This suggests that the relationship between perplexity and predictive power may be mostly driven by the difference in their primary architecture or the amount of data used for training.

This article is an extended presentation of Oh et al. (2021), with additional algorithmic details of the left-corner parser and evaluations of structural parsers and neural LMs as surprisal estimators. These additional evaluations include a quantitative analysis of the effect of model capacity on predictive power for neural LMs, as well as a replication of the main experiments using a different regression method that is sensitive to temporal diffusion. Code used in this work can be found at https://github.com/modelblocks/modelblocks-release and https://github.com/byungdoh/acl21_semproc.

The remainder of this article is structured as follows: Section 2 reviews earlier literature on evaluating neural and structural models of sentence processing; Section 3 provides a formal background on surprisal and left-corner parsing; Section 4 introduces our structural processing model; Sections 5 to 8 outline the regression experiments using data from human subjects; and Section 9 concludes with a discussion of the main findings.

## 2. Related Work

Several recent studies have examined the predictive ability of various neural and structural models on psycholinguistic data using surprisal predictors. Goodkind and Bicknell (2018) compare surprisal-based predictions from a set of *n*-gram, LSTM, and interpolated (LSTM + *n*-gram) LMs. Testing on the Dundee eye-tracking corpus (Kennedy et al., 2003), the authors report a linear relationship between the LM's linguistic quality (measured by perplexity) and its psychometric predictive power (measured by regression model fit).

Wilcox et al. (2020) perform a similar analysis with more model classes, evaluating *n*-gram, LSTM, Transformer, and RNNG models on self-paced reading and eye-tracking data. Each type of LM is trained from scratch on corpora of varying sizes. Their results partially support the linear relationship between perplexity and psychometric predictive power reported in Goodkind and Bicknell (2018), although they note a more exponential relationship at certain intervals. In addition, Wilcox et al. also find that a model's primary architecture affects its psychometric predictive power. When perplexity is held roughly constant, Transformer models tend to make the best reading time and eye-tracking predictions, followed by *n*-gram models, LSTM models, and RNNG models.

Hao et al. (2020) also examine psycholinguistic predictions from Transformer, *n*-gram, and LSTM models, evaluating each on eye-tracking data. Large pretrained Transformers such as GPT-2 (Radford et al., 2019) are tested alongside smaller Transformers trained from scratch. When comparing perplexity and psycholinguistic performance, Hao et al. observe a similar trend across architectures to that reported by Wilcox et al. (2020), with Transformers performing best and LSTMs performing worse compared to *n*-gram models. However, Hao et al. argue that perplexity is flawed as a predictor of psychometric predictive ability, given that perplexity is sensitive to a model's vocabulary size. Instead, they introduce a new metric for evaluating LM performance, Predictability Norm Correlation (PNC), which is defined as the Pearson correlation between surprisal values from a language model and surprisal values measured from human subjects using the Cloze task. Their subsequent evaluation shows a more robust relationship between PNC and psycholinguistic performance than between perplexity and psycholinguistic performance.

Aurnhammer and Frank (2019) compare a set of SRN, LSTM, and Gated Recurrent Unit (GRU; Cho et al., 2014) models, all trained on Section 1 of the English Corpora from the Web (ENCOW; Schäfer, 2015), on their ability to predict self-paced reading times, eye-gaze durations, and N400 measures from electroencephalography (EEG) experiments. They find that as long as the three types of models achieve a similar level of language modeling performance, there is no reliable difference in their predictive power. Merkx and Frank (2021) extend this study by comparing Transformer models against GRU models following similar experimental methods. The Transformer models are found to outperform the GRU models on explaining self-paced reading times and N400 measures but not eye-gaze durations. The authors view this as evidence that human sentence processing may involve cue-based retrieval rather than recurrent processing.

## 3. Background

The experiments presented in this article use surprisal predictors (Shannon, 1948) calculated by an incremental processing model based on a left-corner parser (Johnson-Laird, 1983; van Schijndel et al., 2013). This incremental processing model provides a probabilistic account of sentence processing by making a single lexical attachment decision and a single grammatical attachment decision for each input word.

### 3.1. Surprisal

Surprisal can be defined as the negative log ratio of prefix probabilities of word sequences *w*_1..*t*_ at consecutive time steps *t* − 1 and *t*:


(1) $$\begin{array}{l} \text{S}\left(w_{t}\right)\overset{\text{def}}{=}-\log\frac{\text{P}\left(w_{1..t}\right)}{\text{P}\left(w_{1..t-1}\right)} \end{array}$$

These prefix probabilities can be calculated by marginalizing over the hidden states *q*_*t*_ of the forward probabilities of an incremental processing model:


(2) $$\begin{array}{l} \text{P}\left(w_{1..t}\right)=\underset{q_{t}}{\sum }\text{P}\left(w_{1..t}\;q_{t}\right) \end{array}$$

These forward probabilities are in turn defined recursively using a transition model:


(3) $$\begin{array}{l} \text{P}\left(w_{1..t}\;q_{t}\right)\overset{\text{def}}{=}\underset{q_{t-1}}{\sum }\text{P}\left(w_{t}\;q_{t}|q_{t-1}\right)\cdot \text{P}\left(w_{1..t-1}\;q_{t-1}\right) \end{array}$$

### 3.2. Left-Corner Parsing

Some of the transition models presented in this article are based on a probabilistic left-corner parser (Johnson-Laird, 1983; van Schijndel et al., 2013). Left-corner parsers have been used to model human sentence processing because they define a fixed number of decisions at every time step and also require only a bounded amount of working memory, in keeping with experimental observations of human memory limits (Miller and Isard, 1963). The transition model maintains a distribution over possible working memory store states *q*_*t*_ at every time step *t*, each of which consists of a bounded number *D* of nested derivation fragments $a_{t}^{d}/b_{t}^{d}$. Each derivation fragment spans a part of a derivation tree below some apex node $a_{t}^{d}$ lacking a base node $b_{t}^{d}$ yet to come. Previous work has shown that large annotated corpora such as the Penn Treebank (Marcus et al., 1993) do not require more than *D* = 4 of such fragments (Schuler et al., 2010).

**Figure 1:**
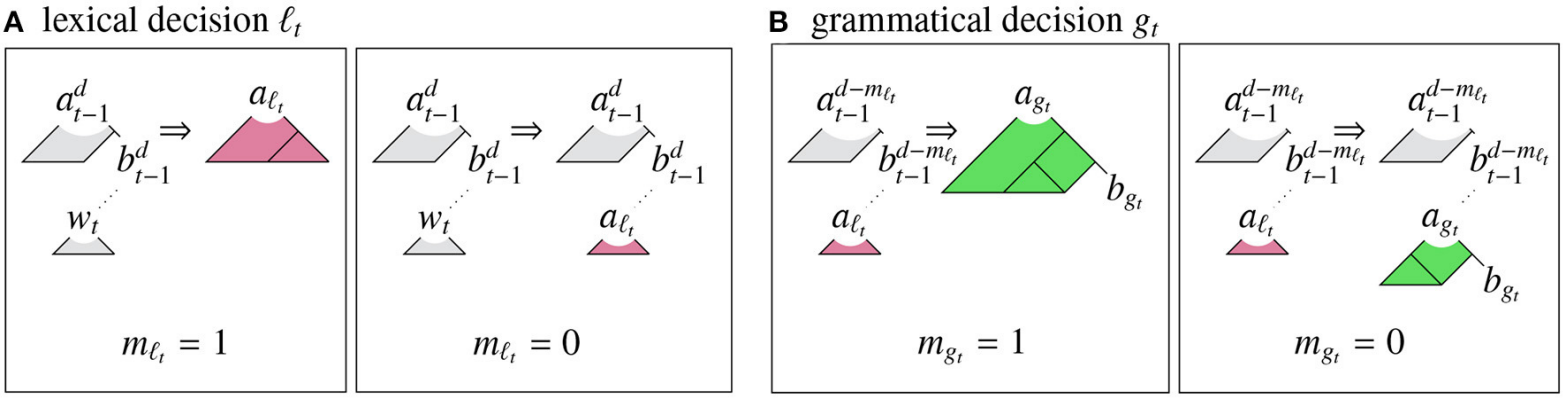
Left-corner parser operations: **(A)** lexical match (*m*_ℓ_*t*__ = 1) and no-match (*m*_ℓ_*t*__ = 0) operations, creating new apex *a*_ℓ_*t*__, and **(B)** grammatical match (*m*_*g*_*t*__ = 1) and no-match (*m*_*g*_*t*__ = 0) operations, creating new apex *a*_*g*_*t*__ and base *b*_*g*_*t*__.

At each time step, a left-corner parsing model generates a new word *w*_*t*_ and a new store state *q*_*t*_ in two phases (see Figure 1). First, it makes a set of *lexical* decisions ℓ_*t*_ regarding whether to use the word to complete the most recent derivation fragment (*match*; *m*_ℓ_*t*__ = 1), or to use the word to create a new preterminal node *a*_ℓ_*t*__ (*no-match*; *m*_ℓ_*t*__ = 0). Subsequently, the model makes a set of *grammatical* decisions *g*_*t*_ regarding whether to use a predicted grammar rule to combine the node constructed in the lexical phase *a*_ℓ_*t*__ with the next most recent derivation fragment (*match*; *m*_*g*_*t*__ = 1), or to use the grammar rule to convert this node into a new derivation fragment *a*_*g*_*t*__/*b*_*g*_*t*__ (*no-match*; *m*_*g*_*t*__ = 0)^1^:


(4) $$\begin{array}{l} \text{P}\left(w_{t}\;q_{t}|q_{t-1}\right)=\underset{\ell_{t},g_{t}}{\sum }\;\text{P}\left(\ell_{t}|q_{t-1}\right)\;\cdot \\ \;\text{P}\left(w_{t}|q_{t-1}\;\ell_{t}\right)\;\cdot \\ \;\text{P}\left(g_{t}|q_{t-1}\;\ell_{t}\;w_{t}\right)\;\cdot \\ \;\text{P}\left(q_{t}|q_{t-1}\;\ell_{t}\;w_{t}\;g_{t}\right) \end{array}$$

Thus, the parser creates a hierarchically organized sequence of derivation fragments and joins these fragments up whenever expectations are satisfied.

In order to update the store state based on the lexical and grammatical decisions, derivation fragments above the most recent nonterminal node are carried forward, and derivation fragments below it are set to null (⊥):


(5) $$\begin{array}{l} \text{P}\left(q_{t}|\ldots \right)\overset{\text{def}}{=}\overset{D}{\underset{d^{\prime }=1}{\prod }}\{\begin{array}{cc} \left[\left[a_{t}^{d^{\prime }},b_{t}^{d^{\prime }}=a_{t-1}^{d^{\prime }},b_{t-1}^{d^{\prime }}\right]\right] & \text{if }d^{\prime }<d\\ \left[\left[a_{t}^{d^{\prime }},b_{t}^{d^{\prime }}=a_{g_{t}},b_{g_{t}}\right]\right] & \text{if }d^{\prime }=d\\ \left[\left[a_{t}^{d^{\prime }},b_{t}^{d^{\prime }}=⊥,⊥\right]\right] & \text{if }d^{\prime }>d \end{array} \end{array}$$

where the indicator function [[φ]] = 1 if φ is true and 0 otherwise, and $d={\text{argmax}}_{d^{\prime }}\left\{a_{t-1}^{d^{\prime }}\neq ⊥\right\}+1-m_{\ell_{t}}-m_{g_{t}}$. Together, these probabilistic decisions generate the *n* unary branches and *n* − 1 binary branches of a parse tree in Chomsky normal form for an *n*-word sentence.

## 4. Structural Processing Model

Unlike the large pretrained neural LMs used in these experiments, the structural processing model is defined in terms of a set of common linguistic abstractions, including

- *Syntax trees* with nodes labeled by *syntactic categories* drawn from a generalized categorial grammar (Ajdukiewicz, 1935; Bar-Hillel, 1953; Bach, 1981; Nguyen et al., 2012),
- *Logical predicates* with arguments signified by associated nodes in the tree, and
- *Morphological rules* which associate transformations in lexical orthography with transformations between syntactic categories of words.

These form the “strong generalizations” in the introduction and conclusion of this article.

### 4.1. Processing Model

**Figure 2:**
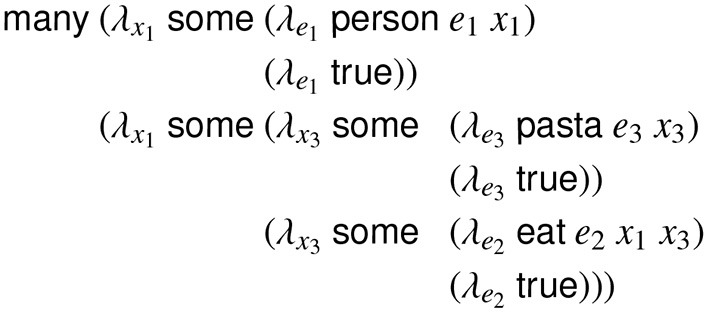
Lambda calculus expression for the propositional content of the sentence. *Many people eat pasta*, using generalized quantifiers over discourse entities and eventualities.

The structural processing model extends the above left-corner parser (Section 3.2) to maintain lemmatized predicate information by augmenting each preterminal, apex, and base node to consist not only of a syntactic category label *c*_*p*_*t*__, $c_{a_{t}^{d}}$, or $c_{b_{t}^{d}}$, but also of a binary *predicate context vector* **h**_*p*_*t*__, ${\text{h}}_{a_{t}^{d}}$, or ${\text{h}}_{b_{t}^{d}}\in {\left\{0,1\right\}}^{K+VK+EK}$, where *K* is the size of the set of predicate contexts and *V* is the maximum valence of any syntactic category^2^, and *E* is the maximum number of non-local arguments (e.g., gap fillers) expressed in any category. Each 0 or 1 element of this vector represents a unique *predicate context*, which consists of a 〈*predicate, role*〉 pair that specifies the content constraints of a node in a predicate-argument structure. These predicate contexts are obtained by reannotating the training corpus using a generalized categorial grammar of English (Nguyen et al., 2012)^3^, which is sensitive to syntactic valence and non-local dependencies. For example, in Figure 2, the variable *e*_2_ (signified by the word *eat*) would have the predicate context EAT_0_ because it is the zeroth (initial) participant of the predication (eat *e*_2_ *x*_1_ *x*_3_)^4^. Similarly, the variable *x*_3_ would have both the predicate context pasta_1_, because it is the first participant (counting from zero) of the predication (pasta *e*_3_ *x*_3_), and the predicate context EAT_2_, because it is the second participant (counting from zero) of the predication (eat *e*_2_ *x*_1_ *x*_3_).

#### 4.1.1. Lexical Decisions

Each lexical decision of the parser includes a match decision *m*_ℓ_*t*__ and decisions about a syntactic category *c*_ℓ_*t*__ and a predicate context vector **h**_ℓ_*t*__ that together specify a preterminal node *p*_ℓ_*t*__. The probability of generating the match decision and the predicate context vector depends on the base node $b_{t-1}^{d}$ of the previous derivation fragment (i.e., its syntactic category and predicate context vector). The first term of Equation (4) can therefore be decomposed into the following:


(6) $$\begin{array}{l} \text{P}\left(\ell_{t}|q_{t-1}\right)=\;\underset{m_{\ell_{t}}{\text{h}}_{\ell_{t}}}{\text{S}\text{OFT}\text{M}\text{AX}}\left({\text{FF}}_{\theta_{\text{L}}}\left[{\delta_{d}}^{⊤},\left[\delta_{c_{b_{t-1}^{d}}}^{⊤},{\text{h}}_{b_{t-1}^{d}}^{⊤}\right]{\text{E}}_{\text{L}}\right]\right)\cdot \\ \;\text{P}\left(c_{\ell_{t}}|q_{t-1}\;m_{\ell_{t}}\;{\text{h}}_{\ell_{t}}\right) \end{array}$$

where FF is a feedforward neural network, and δ_*i*_ is a Kronecker delta vector consisting of a one at element *i* and zeros elsewhere. Depth $d={\text{argmax}}_{d^{\prime }}\left\{a_{t-1}^{d^{\prime }}\neq ⊥\right\}$ is the number of non-null derivation fragments at the previous time step, and **E**_L_ is a matrix of jointly trained dense embeddings for each syntactic category and predicate context. The syntactic category and predicate context vector together define a complete preterminal node *p*_ℓ_*t*__ for use in the word generation model:


(7) $$\begin{array}{l} p_{\ell_{t}}\overset{\text{def}}{=}\{\begin{array}{ll} c_{b_{t-1}^{d}},{\text{h}}_{b_{t-1}^{d}}+{\text{h}}_{\ell_{t}} & \text{if }m_{\ell_{t}}=1\\ c_{\ell_{t}},{\text{h}}_{\ell_{t}} & \text{if }m_{\ell_{t}}=0 \end{array} \end{array}$$

and a new apex node *a*_ℓ_*t*__ for use in the grammatical decision model:


(8) $$\begin{array}{l} a_{\ell_{t}}\overset{\text{def}}{=}\{\begin{array}{ll} c_{a_{t-1}^{d}},{\text{h}}_{a_{t-1}^{d}}+{\text{Z}}_{t-1}\;{\text{h}}_{p_{\ell_{t}}} & \text{if }m_{\ell_{t}}=1\\ p_{\ell_{t}} & \text{if }m_{\ell_{t}}=0 \end{array} \end{array}$$

where **Z**_*t*_ propagates predicate contexts from right progeny back up to apex nodes (see Equation 12 below).

#### 4.1.2. Grammatical Decisions

Each grammatical decision includes a match decision *m*_*g*_*t*__ and decisions about a pair of syntactic category labels *c*_*g*_*t*__ and $c_{g_{t}}^{\prime }$, as well as a predicate context composition operator *o*_*g*_*t*__, which governs how the newly generated predicate context vector **h**_ℓ_*t*__ is propagated through its new derivation fragment *a*_*g*_*t*__/*b*_*g*_*t*__. The probability of generating the match decision and the composition operators depends on the base node $b_{t-1}^{d-m_{\ell_{t}}}$ of the previous derivation fragment and the apex node *a*_ℓ_*t*__ from the current lexical decision (i.e., their syntactic categories and predicate context vectors). The third term of Equation (4) can accordingly be decomposed into the following:


(9) $$\begin{array}{l} \text{P}\left(g_{t}|q_{t-1}\;\ell_{t}\;w_{t}\right)\\ =\underset{m_{g_{t}}o_{g_{t}}}{\text{S}\text{OFT}\text{M}\text{AX}}\left({\text{FF}}_{\theta_{\text{G}}}\left[{\delta_{d}}^{⊤},\left[\delta_{c_{b_{t-1}^{d-m_{\ell_{t}}}}}^{⊤},{\text{h}}_{b_{t-1}^{d-m_{\ell_{t}}}}^{⊤},\delta_{c_{a_{\ell_{t}}}}^{⊤},{\text{h}}_{a_{\ell_{t}}}^{⊤}\right]{\text{E}}_{\text{G}}\right]\right)\;\cdot \\ \;\text{P}\left(c_{g_{t}}|q_{t-1}\;\ell_{t}\;w_{t}\;m_{g_{t}}\;o_{g_{t}}\right)\cdot \;\text{P}\left(c_{g_{t}}^{\prime }|q_{t-1}\;\ell_{t}\;w_{t}\;m_{g_{t}}\;o_{g_{t}}\;c_{g_{t}}\right) \end{array}$$

where **E**_G_ is a matrix of jointly trained dense embeddings for each syntactic category and predicate context. The composition operators are associated with sparse composition matrices **A**_*o*_*g*_*t*___, defined in Appendix A, which can be used to compose predicate context vectors associated with the apex node *a*_*g*_*t*__:


(10) $$\begin{array}{l} a_{g_{t}}\overset{\text{def}}{=}\{\begin{array}{ll} c_{a_{t-1}^{d-m_{\ell_{t}}}},{\text{h}}_{a_{t-1}^{d-m_{\ell_{t}}}}+{\text{Z}}_{t-1}\;{\text{A}}_{o_{g_{t}}}^{⊤}{\text{h}}_{a_{\ell_{t}}} & \text{if }m_{g_{t}}=1\\ c_{g_{t}},{\text{A}}_{o_{g_{t}}}^{⊤}{\text{h}}_{a_{\ell_{t}}} & \text{if }m_{g_{t}}=0 \end{array} \end{array}$$

and sparse composition matrices **B**_*o*_*g*_*t*___, also defined in Appendix A, which can be used to compose predicate context vectors associated with the base node *b*_*g*_*t*__:


(11) $$\begin{array}{l} b_{g_{t}}\overset{\text{def}}{=}\{\begin{array}{ll} c_{g_{t}}^{\prime },{\text{B}}_{o_{g_{t}}}{\left[{{\text{h}}_{b_{t-1}^{d-m_{\ell_{t}}}}}^{⊤},{{\text{h}}_{a_{\ell_{t}}}}^{⊤}\right]}^{⊤} & \text{if }m_{g_{t}}=1\\ c_{g_{t}}^{\prime },{\text{B}}_{o_{g_{t}}}{\left[0^{⊤},{{\text{h}}_{a_{\ell_{t}}}}^{⊤}\right]}^{⊤} & \text{if }m_{g_{t}}=0 \end{array} \end{array}$$

Matrix **Z**_*t*_ propagates predicate contexts from right progeny back up to apex nodes^5^:


(12) $$\begin{array}{l} {\text{Z}}_{t}\overset{\text{def}}{=}\{\begin{array}{ll} {\text{Z}}_{t-1}\;\left[0^{H\times H},{\text{I}}^{H\times H}\right]\;{{\text{B}}_{o_{g_{t}}}}^{⊤} & \text{if }m_{g_{t}}=1\\ \left[0^{H\times H},{\text{I}}^{H\times H}\right]\;{{\text{B}}_{o_{g_{t}}}}^{⊤} & \text{if }m_{g_{t}}=0 \end{array} \end{array}$$

### 4.2. Character-Based Morphological Word Model

A character-based morphological word model applies a morphological rule *r*_*t*_ to a lemma *x*_*t*_ to generate an inflected form *w*_*t*_. The set of rules model affixation through string substitution and are inverses of lemmatization rules that are used to derive predicates in the generalized categorial grammar annotation (Nguyen et al., 2012). For example, the rule %ay→%aid can apply to the word *say* to derive its past tense form *said*. There are around 600 such rules that account for inflection in Sections 02 to 21 of the Wall Street Journal corpus of the Penn Treebank (Marcus et al., 1993), which includes an identity rule for words in bare form and a “no semantics” rule for generating certain function words.

For an observed input word *w*_*t*_, the model first generates a list of 〈*x*_*t*_, *r*_*t*_〉 pairs that deterministically generate *w*_*t*_. This allows the model to capture morphological regularity and estimate how expected a word form is given its predicted syntactic category and predicate context, which have been generated as part of the preceding lexical decision. In addition, this lets the model hypothesize the underlying morphological structure of out-of-vocabulary words and assign probabilities to them. The second term of Equation (4) can thus be decomposed into the following:


(13) $$\begin{array}{l} \text{P}\left(w_{t}|q_{t-1}\;\ell_{t}\right)=\underset{x_{t},r_{t}}{\sum }\;\text{P}\left(x_{t}|q_{t-1}\;\ell_{t}\right)\;\cdot \\ \;\text{P}\left(r_{t}|q_{t-1}\;\ell_{t}\;x_{t}\right)\;\cdot \\ \;\text{P}\left(w_{t}|q_{t-1}\;\ell_{t}\;x_{t}\;r_{t}\right) \end{array}$$

The probability of generating the lemma sequence depends on the syntactic category *c*_*p*_ℓ_*t*___ and predicate context **h**_ℓ_*t*__ resulting from the preceding lexical decision ℓ_*t*_:


(14) $$\begin{array}{l} \text{P}\left(x_{t}|q_{t-1}\;\ell_{t}\right)=\underset{i}{\prod }\underset{x_{t,i}}{\text{S}\text{OFT}\text{M}\text{AX}}\left({\text{W}}_{\text{X}}{\text{x}}_{t,i}+{\text{b}}_{\text{X}}\right) \end{array}$$

where *x*_*t*,1_, *x*_*t*,2_, …, *x*_*t,I*_ is the character sequence of lemma *x*_*t*_, with *x*_*t*,1_ = 〈*s*〉 and *x*_*t,I*_ = 〈*e*〉 as special start and end characters. **W**_X_ and **b**_X_ are, respectively, a weight matrix and bias vector of a softmax classifier. A recurrent neural network (RNN) calculates a hidden state **x**_*t,i*_ for each character from an input vector at that time step and the hidden state after the previous character **x**_*t,i*−1_:


(15) $$\begin{array}{l} {\text{x}}_{t,i}={\text{RNN}}_{\theta_{\text{X}}}\left(\left[\delta_{c_{p_{\ell_{t}}}}^{⊤},{\text{h}}_{\ell_{t}}^{⊤},\delta_{x_{t,i}}^{⊤}\right]\;{\text{E}}_{\text{X}},{\text{x}}_{t,i-1}^{⊤}\right) \end{array}$$

where **E**_X_ is a matrix of jointly trained dense embeddings for each syntactic category, predicate context, and character.

Subsequently, the probability of applying a particular morphological rule to the generated lemma depends on the syntactic category *c*_*p*_ℓ_*t*___ and predicate context **h**_ℓ_*t*__ from the preceding lexical decision as well as the character sequence of the lemma:


(16) $$\begin{array}{l} \text{P}\left(r_{t}|q_{t-1}\;\ell_{t}\;x_{t}\right)=\underset{r_{t}}{\text{S}\text{OFT}\text{M}\text{AX}}\left({\text{W}}_{\text{R}}{\text{r}}_{t,I}+{\text{b}}_{\text{R}}\right) \end{array}$$

Here, **W**_R_ and **b**_R_ are, respectively, a weight matrix and bias vector of a softmax classifier. **r**_*t,I*_ is the last hidden state of an RNN that takes as input the syntactic category, predicate context, and character sequence of the lemma *x*_*t*, 2_, *x*_*t*, 3_, …, *x*_*t,I*−1_ without the special start and end characters:


(17) $$\begin{array}{l} {\text{r}}_{t,i}={\text{RNN}}_{\theta_{\text{R}}}\left(\left[\delta_{c_{p_{\ell_{t}}}}^{⊤},{\text{h}}_{\ell_{t}}^{⊤},\delta_{x_{t,i}}^{⊤}\right]\;{\text{E}}_{\text{R}},{\text{r}}_{t,i-1}^{⊤}\right) \end{array}$$

where **E**_R_ is a matrix of jointly trained dense embeddings for each syntactic category, predicate context, and character.

Finally, as the model calculates probabilities only for 〈*x*_*t*_, *r*_*t*_〉 pairs that deterministically generate *w*_*t*_, the word probability conditioned on these variables P(*w*_*t*_ ∣ *q*_*t*−1_ ℓ_*t*_ *x*_*t*_ *r*_*t*_) = 1.

## 5. Experiment 1: Predictive Power of Surprisal Estimates

In order to compare the predictive power of surprisal estimates from structural parsers and LMs, regression models containing common baseline predictors and a surprisal predictor were fitted to self-paced reading times, eye-gaze durations, and blood oxygenation level-dependent signals collected during naturalistic language processing. For self-paced reading times and eye-gaze durations that were measured at the word level, linear mixed-effects models were fitted to the response data. In contrast, for blood oxygenation level-dependent signals that were measured in fixed-time intervals, the novel statistical framework of continuous-time deconvolutional regression (CDR; Shain and Schuler, 2021) was employed. As CDR allows the data-driven estimation of continuous impulse response functions from variably spaced linguistic input, it is more appropriate for modeling fMRI responses, which are typically measured in fixed time intervals. To compare the predictive power of surprisal estimates from different models on equal footing, we calculated the increase in log-likelihood (ΔLL) to a baseline regression model as a result of including a surprisal predictor, following recent work (Goodkind and Bicknell, 2018; Aurnhammer and Frank, 2019; Hao et al., 2020; Wilcox et al., 2020).

### 5.1. Response Data

#### 5.1.1. Self-Paced Reading Times

The first experiment described in this article used the Natural Stories Corpus (Futrell et al., 2021), which contains self-paced reading times from 181 subjects that read 10 naturalistic stories consisting of 10,245 tokens. The data were filtered to exclude observations corresponding to sentence-initial and sentence-final words, observations from subjects who answered fewer than four comprehension questions correctly, and observations with durations shorter than 100 ms or longer than 3,000 ms. This resulted in a total of 770,102 observations, which were subsequently partitioned into an exploratory set of 384,905 observations and a held-out set of 385,197 observations^6^. The partitioning allows model selection (e.g., making decisions about baseline predictors and random effects structure) to be conducted on the exploratory set and a single hypothesis test to be conducted on the held-out set, thus eliminating the need for multiple trials correction. All observations were log-transformed prior to model fitting.

#### 5.1.2. Eye-Gaze Durations

Additionally, the set of go-past durations from the Dundee Corpus (Kennedy et al., 2003) provided the response variable for the regression models. The Dundee Corpus contains eye-gaze durations from 10 subjects that read 67 newspaper editorials consisting of 51,501 tokens. The data were filtered to exclude unfixated words, words following saccades longer than four words, and words at starts and ends of sentences, screens, documents, and lines. This resulted in a total of 195,507 observations, which were subsequently partitioned into an exploratory set of 98,115 observations and a held-out set of 97,392 observations^7^. All observations were log-transformed prior to model fitting.

#### 5.1.3. Blood Oxygenation Level-Dependent Signals

Finally, the time series of blood oxygenation level-dependent (BOLD) signals in the language network, which were identified using functional magnetic resonance imaging (fMRI), were analyzed. This experiment used the same fMRI data used by Shain et al. (2020), which were collected at a fixed-time interval of every 2 s from 78 subjects that listened to a recorded version of the Natural Stories Corpus. The functional regions of interest (fROI) corresponding to the domain-specific language network were identified for each subject based on the results of a localizer task that they conducted. This resulted in a total of 194,859 observations, which were subsequently partitioned into an exploratory set of 98,115 observations and a held-out set of 96,744 observations^8^.

### 5.2. Predictors

#### 5.2.1. Baseline Predictors

For each dataset, a set of baseline predictors that capture low-level cognitive processing were included in all regression models.

- Self-paced reading times (Futrell et al., 2021): word length measured in characters, index of word position within each sentence
- Eye-gaze durations (Kennedy et al., 2003): word length measured in characters, index of word position within each sentence, saccade length, whether or not the previous word was fixated
- BOLD signals (Shain et al., 2020): index of fMRI sample within the current scan, the deconvolutional intercept which captures the influence of stimulus timing, whether or not the word is at the end of sentence, duration of pause between the current word and the next word.

#### 5.2.2. Surprisal Estimates

For regression modeling, surprisal estimates were also calculated from all models evaluated in this experiment. This includes the structural processing model described in Section 4, which was trained on a generalized categorial grammar (GCG; Nguyen et al., 2012) reannotation of Sections 02 to 21 of the Wall Street Journal (WSJ) corpus of the Penn Treebank (Marcus et al., 1993). Beam search decoding with a beam size of 5,000 was used to estimate prefix probabilities and by-word surprisal for this model^9^.

Additionally, in order to assess the contribution of linguistic abstractions, two ablated variants of the above structural processing model were trained and evaluated.

−*cat*: This variant ablates the contribution of syntactic category labels to the lexical and grammatical decisions by zeroing out their associated dense embeddings in Equations (6) and (9).
−*morph*: This variant ablates the contribution of the character-based morphological word model by calculating the word generation probabilities (i.e., Equation 13) using relative frequency estimation.

Finally, various incremental parsers and pretrained LMs were used to calculate surprisal estimates at each word.

- *RNNG* (Dyer et al., 2016; Hale et al., 2018): An LSTM-based model with explicit phrase structure, trained on Sections 02 to 21 of the WSJ corpus.
- *vSLC* (van Schijndel et al., 2013): A left-corner parser based on a PCFG with subcategorized syntactic categories (Petrov et al., 2006), trained on a generalized categorial grammar reannotation of Sections 02 to 21 of the WSJ corpus.
- *JLC* (Jin and Schuler, 2020): A neural left-corner parser based on stack LSTMs (Dyer et al., 2015), trained on Sections 02 to 21 of the WSJ corpus.
- *5-gram* (Heafield et al., 2013): A 5-gram language model with modified Kneser-Ney smoothing trained on ~3B tokens of the English Gigaword Corpus (Parker et al., 2009).
- *GLSTM* (Gulordava et al., 2018): A two-layer LSTM model trained on ~80M tokens of the English Wikipedia.
- *JLSTM* (Jozefowicz et al., 2016): A two-layer LSTM model with CNN character inputs trained on ~800M tokens of the One Billion Word Benchmark (Chelba et al., 2014).
- *GPT2XL* (Radford et al., 2019): GPT-2 XL, a 48-layer decoder-only autoregressive Transformer model trained on ~8B tokens of the WebText dataset.

### 5.3. Procedures

To calculate the increase in log-likelihood (ΔLL) attributable to each surprisal predictor, a *baseline* regression model containing only the baseline predictors (Section 5.2.1) was first fitted to the held-out set of each dataset. For self-paced reading times and eye-gaze durations which are by-word response measures, linear mixed-effects (LME) models were fitted using lme4 (Bates et al., 2015). All baseline predictors were centered and scaled prior to model fitting, and the baseline LME models included by-subject random slopes for all fixed effects and random intercepts for each word and subject-sentence interaction. For BOLD signals that were measured in fixed-time intervals, there is a temporal misalignment between the linguistic input (i.e., words that are variably spaced) and the response measures (i.e., BOLD signals measured at fixed-time intervals), making them less appropriate to model using LME regression. To overcome this issue without arbitrarily coercing the data, the novel statistical framework of continuous-time deconvolutional regression^10^ (CDR; Shain and Schuler, 2021) was employed to estimate continuous hemodynamic response functions (HRF). Following Shain et al. (2020), the baseline CDR model assumed the two-parameter HRF based on the double-gamma canonical HRF (Lindquist et al., 2009). Furthermore, the two parameters of the HRF were tied across predictors, modeling the assumption that the shape of the blood oxygenation response to neural activity is identical in a given region. However, to allow the HRFs to have differing amplitudes, a coefficient that rescales the HRF was estimated for each predictor. The “index of fMRI sample” and “duration of pause” baseline predictors were scaled, and the baseline CDR model also included a by-fROI random effect for the amplitude coefficient and a by-subject random intercept.

Subsequently, *full* regression models that include one surprisal predictor (Section 5.2.2) on top of the baseline regression model were fitted to the held-out set of each dataset. For self-paced reading times and eye-gaze durations, the surprisal predictor was scaled and centered, and its by-subject random slopes were included in the full LME model. Similarly, for BOLD signals, the surprisal predictor was centered, and its by-fROI random effect for the amplitude coefficient was included in the full CDR model. After all the regression models were fitted, ΔLL was calculated by subtracting the log-likelihood of the baseline model from that of a full regression model. This resulted in ΔLL measures for all incremental parsers and LMs on each dataset. Additionally, in order to examine whether any of the models fail to generalize across domains, their perplexity on the entire Natural Stories and Dundee corpora was also calculated.

### 5.4. Results

**Figure 3:**
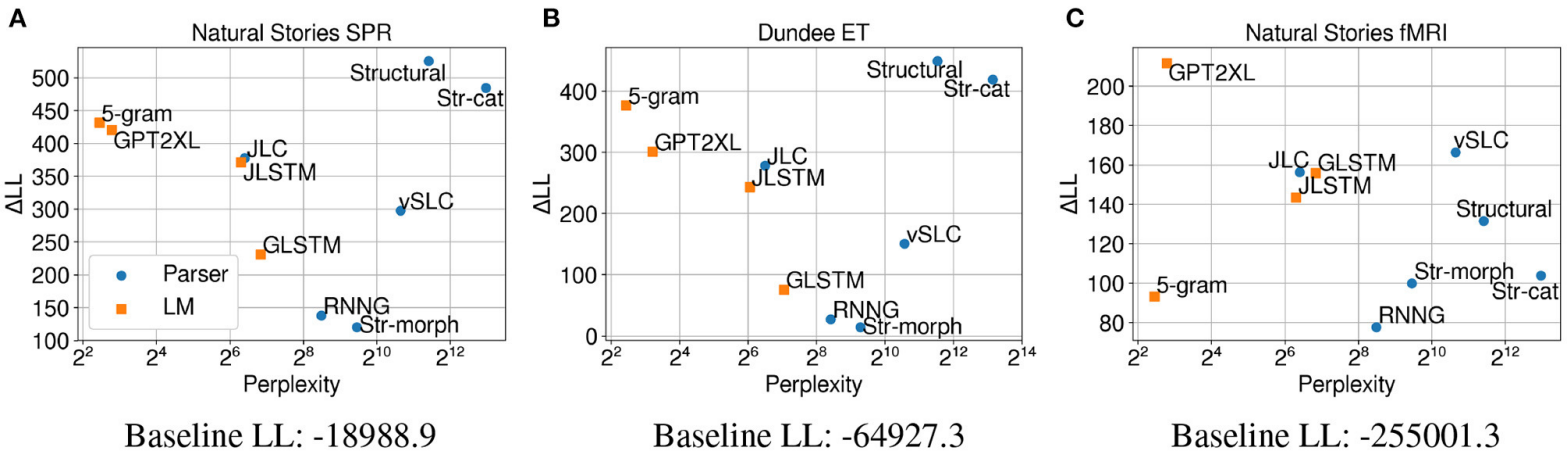
Perplexity measures from each model, and improvements in LMER **(A,B)** and CDR **(C)** model log-likelihood from including each surprisal estimate on **(A)** Natural Stories self-paced reading data, **(B)** Dundee eye-tracking data, and **(C)** Natural Stories fMRI data. The difference in by-item squared error between the *Structural* and *GPT2XL* models is significant at *p* < 0.05 level for all three datasets.

The results in Figure 3A show that surprisal from our structural model (*Structural*) made the biggest contribution to regression model fit compared to surprisal from other models on self-paced reading times. This finding, despite the fact that the pretrained LMs were trained on much larger datasets and also show lower perplexities on test data^11^, suggests that this model may provide a more humanlike account of processing difficulty. In other words, the strong generalizations that are made by the structural model seem to help predict humanlike processing costs that manifest in self-paced reading times even when the amount of training data is limited. Performance of the surprisal predictors from ablated variants of the *Structural* model shows that the character-based morphological word model makes an especially large contribution to regression model fit, which may suggest a larger role of morphology and subword information in sentence processing. Additionally, the results show that although parsers like *Structural* and *vSLC* deviate from this pattern, there is generally a monotonic relationship between the test perplexity and the predictive power of the models (Goodkind and Bicknell, 2018; Wilcox et al., 2020). Most notably, the *5-gram* model outperformed the neural LMs in terms of both perplexity and ΔLL. This is most likely due to the fact that the model was trained on much more data (~3B tokens) compared to the LSTM models (~80M and ~800M tokens, respectively) and that it employs modified Kneser-Ney smoothing, which allows lower perplexity to be achieved on words in the context of out-of-vocabulary words.

Results from regression models fitted on eye-gaze durations (Figure 3B) show a very similar trend to self-paced reading times in terms of both perplexity and ΔLL, although the contribution of surprisal predictors in comparison to the baseline regression model is weaker. This provides further support for the observation that the strong linguistic generalizations that are not explicitly made by the LMs do indeed help predict humanlike processing costs. Moreover, the similar trend across the two datasets may indicate that latency-based measures like self-paced reading times and eye-gaze durations capture similar aspects of processing difficulty.

However, the regression models fitted on BOLD signals demonstrate a very different trend (Figure 3C), with surprisal from *GPT2XL* making the biggest contribution to model fit in comparison to surprisal from other models. Most notably, in contrast to self-paced reading times and eye-gaze durations, surprisal estimates from *Structural* and *5-gram* models did not contribute as much to model fit on fMRI data, with a ΔLL lower than those of the LSTM models. This differential contribution of surprisal estimates across datasets suggests that latency-based measures and blood oxygenation levels may be sensitive to different aspects of online processing difficulty.

## 6. Experiment 2: Influence of Model Capacity

The previous experiment revealed that at least for the neural LMs, there is a monotonic relationship between perplexity and predictive power on latency-based measures of comprehension difficulty. Although evaluating “off-the-shelf” LMs that have been shown to be effective allows them to be examined in their most authentic setting without the need of expensive training procedures, this methodology leaves some variables uncontrolled, such as the primary architecture (e.g., Transformers or LSTMs), model capacity, or the training data used. This experiment aims to bring under control the primary architecture as well as the training data associated with LMs by evaluating the perplexity and predictive power of different variants of GPT-2 models, which differ only in terms of model capacity (i.e., number of layers and parameters). To this end, following similar procedures as Experiment 1, surprisal estimates from different variants of GPT-2 models were regressed to self-paced reading times, eye-gaze durations, and BOLD signals to examine their ability to predict behavioral and neural measures.

### 6.1. Procedures

To calculate the ΔLL measure for each GPT-2 surprisal predictor, the same baseline regression models containing the baseline predictors outlined in Section 5.2.1 were adapted from Experiment 1. Subsequently, in order to fit full regression models that include one surprisal predictor on top of the baseline regression model, surprisal estimates from the following GPT-2 models (Radford et al., 2019) that were pretrained on ~8B tokens of the WebText dataset were calculated.

- GPT-2 Small, with 12 layers and ~124M parameters.
- GPT-2 Medium, with 24 layers and ~355M parameters.
- GPT-2 Large, with 36 layers and ~774M parameters.
- GPT-2 XL, with 48 layers and ~1558M parameters.

Similarly to Experiment 1, LME models that contain each of these surprisal predictors were fitted to the held-out set of self-paced reading times and eye-gaze durations using lme4 (Bates et al., 2015). All predictors were centered and scaled prior to model fitting, and the LME models included by-subject random slopes for all fixed effects and random intercepts for each word and subject-sentence interaction. Additionally, CDR models assuming the two-parameter double-gamma canonical HRF were fitted to the held-out set of BOLD signals. Again, the two parameters of the HRF were tied across predictors, but the HRFs were allowed to have differing amplitudes by jointly estimating a coefficient that rescales the HRF for each predictor. The “index of fMRI sample,” “duration of pause,” and surprisal predictors were scaled, and the CDR models also included a by-fROI random effect for the amplitude coefficient and a by-subject random intercept. After all the regression models were fitted, ΔLL for each GPT-2 model was calculated by subtracting the log-likelihood of the baseline model from that of the full regression model which contains its surprisal estimates. To further examine the relationship between perplexity and predictive power, their perplexity on the entire Natural Stories and Dundee corpora was also calculated.

### 6.2. Results

**Figure 4:**
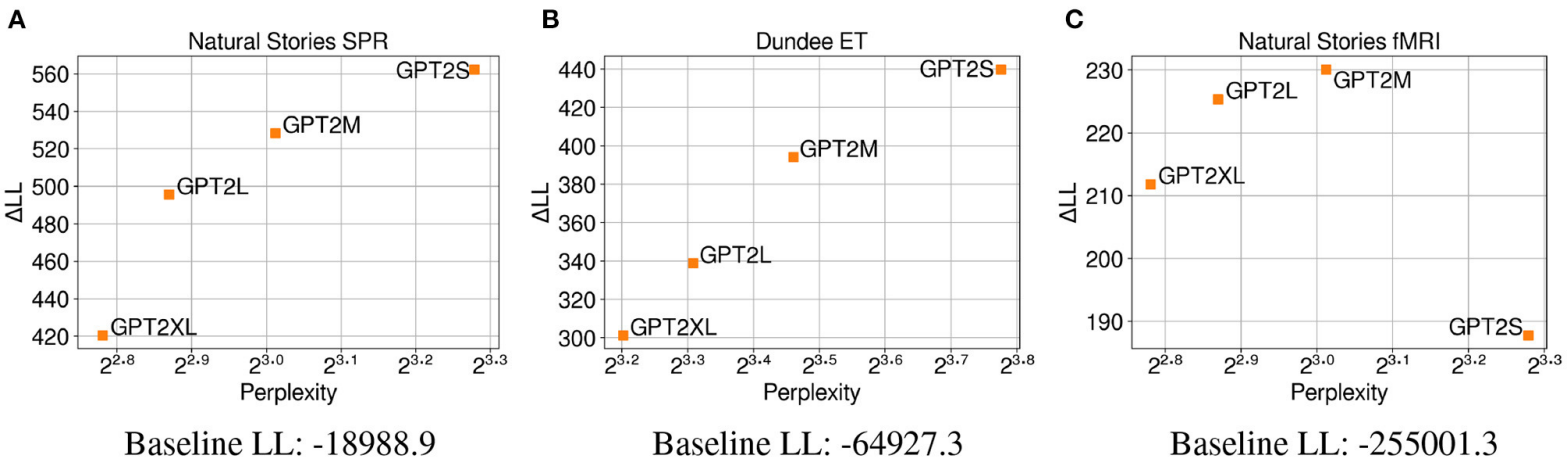
Perplexity measures from each GPT-2 model, and improvements in LMER **(A,B)** and CDR **(C)** model log-likelihood from including each surprisal estimate on **(A)** Natural Stories self-paced reading data, **(B)** Dundee eye-tracking data, and **(C)** Natural Stories fMRI data. The difference in by-item squared error between the *GPT2S* and *GPT2M* models is significant at *p* < 0.05 level for the self-paced reading and eye-tracking data, and the difference in by-item squared error between the *GPT2M* and *GPT2XL* models is significant at *p* < 0.05 level for the fMRI data.

The results in Figure 4A demonstrate that surprisal from GPT-2 Small (*GPT2S*), which has the least number of parameters, made the biggest contribution to regression model fit on self-paced reading times compared to surprisal from larger GPT-2 models that have more parameters. Contrary to the findings of the previous experiment that showed a negative correlation between test perplexity and predictive power, a positive correlation is observed between these two variables from the GPT-2 models that were examined. This may indicate that the trend observed in Experiment 1, where neural LMs with lower perplexity predicted latency-based measures more accurately, may be driven more by the difference in their primary architecture or the amount of data used for training, rather than their model capacity. Additionally, these results may suggest that when the training data is held constant, neural LMs are able to make accurate predictions about the upcoming word while relying less on humanlike generalizations as their capacity increases. In other words, the larger LMs may be able to effectively condition on a much larger context window to make their predictions, while human reading times may be influenced more by a smaller context window. As with Experiment 1, the results from regression models fitted on eye-gaze durations (Figure 4B) show a very similar trend, providing further evidence for the positive relationship between perplexity and predictive power observed on self-paced reading times. Again, the similar trend in perplexity and ΔLL across the two datasets may indicate that latency-based measures capture similar aspects of processing difficulty.

In contrast, the regression models fitted on BOLD signals do not show a clear relationship between perplexity and ΔLL (Figure 4C), with surprisal from *GPT2M* making the biggest contribution to model fit and that from *GPT2S* making the smallest contribution to model fit. Such lack of the pattern observed in latency-based measures could be attributed to the possibility that latency-based measures and blood oxygenation levels are sensitive to different aspects of online processing difficulty, as noted in Experiment 1. Additionally, the fMRI data seems to be noisier in general, as can be seen by the smaller overall contribution of surprisal predictors in comparison to the baseline log-likelihood for the BOLD signals.

## 7. Experiment 3: Replication Using Continuous-Time Deconvolutional Regression

The previous two experiments used LME regression to compare the predictive quality of surprisal estimates from structural parsers and LMs on latency-based measures of comprehension difficulty (i.e., self-paced reading times and eye-gaze durations). Although the use of LME regression is popular in psycholinguistic modeling, it is limited in that it is unable to capture the lingering influence of the *current* predictor on *future* response measures (i.e., temporal diffusion). In the context of latency-based measures, this means that LME models cannot usually take into account the delay in processing that may be caused *after* processing an unusually difficult word. One common approach taken to address this issue is to include “spillover” variants of predictors from preceding words (Rayner et al., 1983; Vasishth, 2006). However, including multiple spillover variants of the same predictor often leads to identifiability issues in LME regression (Shain and Schuler, 2021). Additionally, even spillover predictors may not be able to capture the long-range influence of the input if it falls out of the “spillover window.” This experiment aims to mitigate these drawbacks of LME regression used in the previous experiments by replicating the analysis of latency-based measures using continuous-time deconvolutional regression (CDR; Shain and Schuler, 2021), which allows the data-driven estimation of continuous impulse response functions. To this end, the LME regression analyses of Experiments 1 and 2 were replicated using CDR, following the same protocol of fitting baseline and full regression models and calculating the difference in their log-likelihoods (ΔLL).

### 7.1. Procedures

For both self-paced reading times and eye-gaze durations, baseline CDR models were fitted to the held-out set using the baseline predictors described in Section 5.2.1. In addition, the index of word position within each document^12^ and the deconvolutional intercept that captures the influence of stimulus timing were also included as a baseline predictors. Following Shain and Schuler (2018), the baseline CDR models assumed the three-parameter ShiftedGamma IRF. The “index of word position within each document” and “index of word position within each sentence” predictors were scaled, and the “word length in characters” and “saccade length” predictors were both centered and scaled. The baseline CDR models also included a by-subject random effect for all predictors.

In order to fit full models that include one surprisal predictor on top of the baseline model, surprisal estimates from the parsers and LMs (Section 5.2.2) as well as different variants of the pretrained GPT-2 models (Section 6.1) were calculated. Subsequently, CDR models that contain each of these surprisal predictors were fitted to the held-out set of self-paced reading times and eye-gaze durations. All surprisal predictors were scaled prior to model fitting, and the full CDR models also included a by-subject random effect for the surprisal predictor. After all the regression models were fitted, ΔLL for each model was calculated by subtracting the log-likelihood of the baseline model from that of a full regression model that contains its surprisal estimates.

### 7.2. Results

**Figure 5:**
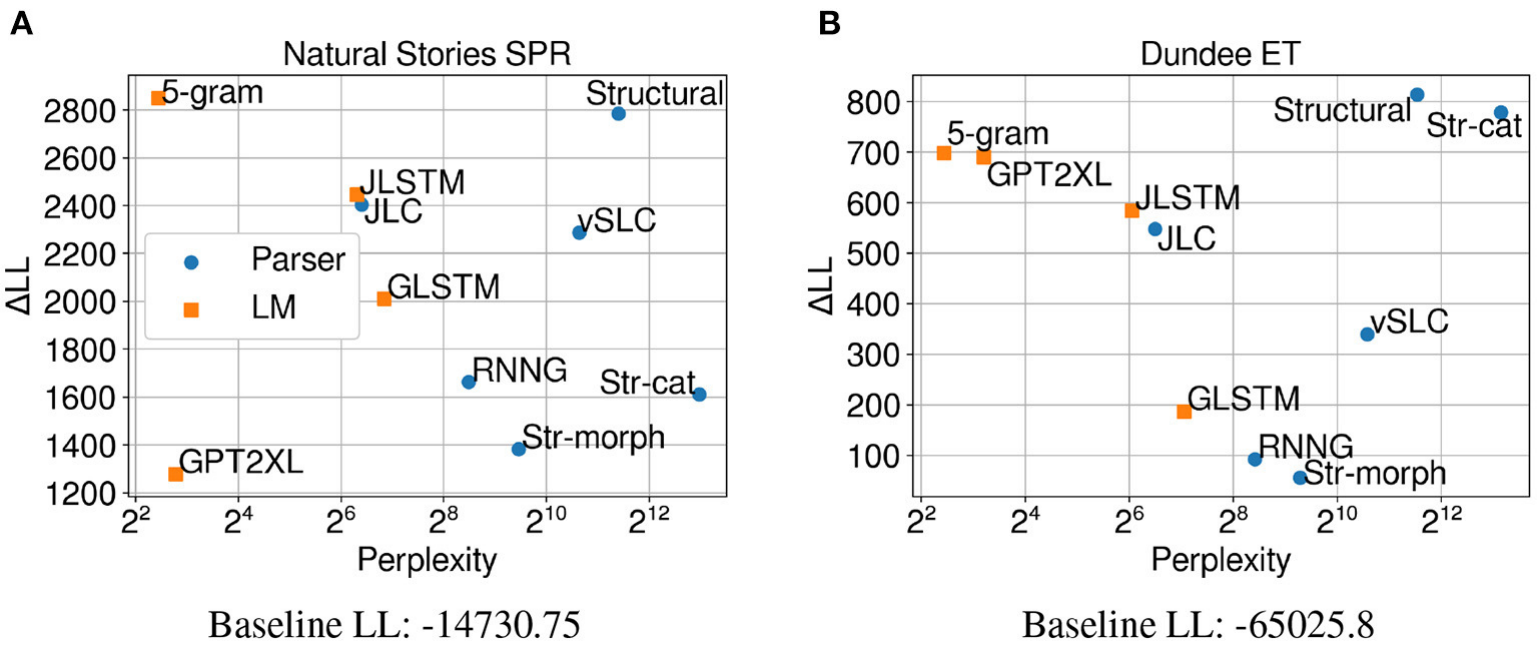
Perplexity measures from each model, and improvements in CDR model log-likelihood from including each surprisal estimate on **(A)** Natural Stories self-paced reading data and **(B)** Dundee eye-tracking data. The difference in by-item squared error between the *Structural* and *GPT2XL* models is significant at *p* < 0.05 level for both datasets.

Figure 5 shows that on both self-paced reading times and eye-gaze durations, using CDR results in higher ΔLL measures for all evaluated models compared to the results using LME regression in Figure 3. This indicates the usefulness of CDR in capturing the lingering influence of surprisal to better explain latency-based measures.

On self-paced reading times, the ΔLL measures from individual models in Figure 5A show a different trend from the LME regression results in Figure 3A. More specifically, surprisal from the *5-gram* model made the biggest contribution to regression model fit, outperforming surprisal from other models in predicting self-paced reading times. Although the strong predictive power of 5-gram surprisal is less expected, one fundamental difference between the *5-gram* model and other models is that it has the shortest context window (i.e., ≤ 4 words due to Kneser-Ney smoothing) among all models. This would result in by-word surprisal estimates that depend especially strongly on the local context, which may provide orthogonal information to the CDR model that considers a sequence of surprisal predictors to make its predictions. Among the neural LMs, the *JLSTM* model now outperforms the others, including the largest GPT-2 model (*GPT2XL*). Again, it may be that using CDR to explicitly condition on previous surprisal values is less beneficial for the Transformer-based GPT-2 models, which may already be incorporating lossless representations of the previous context into their surprisal estimates through their self-attention mechanism.

Among the parsers, the biggest difference is observed for the *Str-cat* variant, which shows predictive power close to the *Structural* model when LME regression is utilized, but is outperformed by all other parsers when CDR is used instead. Although the exact reason behind this phenomenon is unclear, it may be that ablating syntactic category information leads to surprisal estimates that are more faithful to the current word, making them more appropriate for LME regression. Parsers like the *Structural* model and the *JLC* model still outperform neural LMs that were trained on much larger datasets, which further suggests the importance of strong linguistic generalizations in providing a humanlike account of processing difficulty.

CDR models fitted on eye-gaze durations (Figure 5B) show a very similar trend to the LME models (Figure 3B) in terms of both perplexity and ΔLL, although the *JLSTM* model now slightly outperforms the *JLC* model. This similarity between CDR and LME modeling suggests that the lingering influence of previous words may not be as strong as it is on self-paced reading times. Another possibility for this is that useful information about the preceding words is already being captured by the two baseline predictors, “saccade length” and “previous word was fixated,” which are included in both the CDR and LME models.

**Figure 6:**
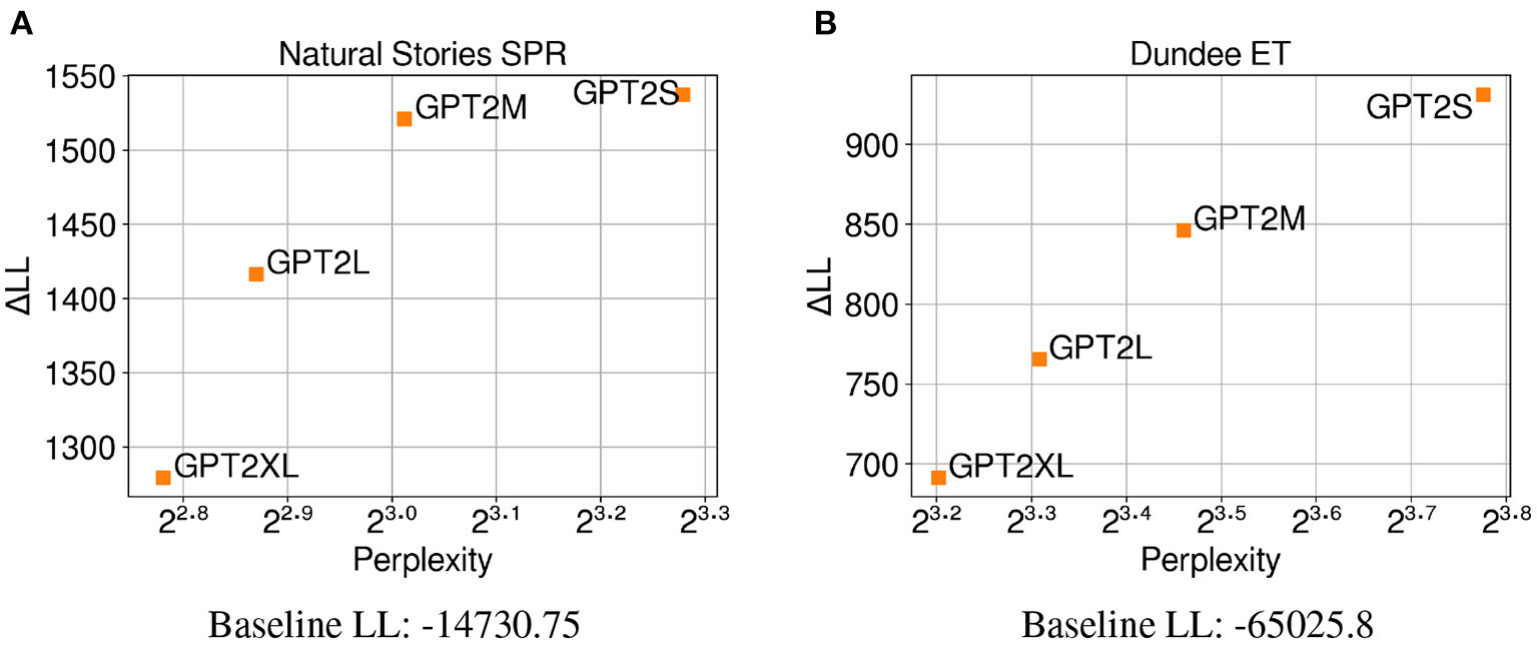
Perplexity measures from each GPT-2 model, and improvements in CDR model log-likelihood from including each surprisal estimate on **(A)** Natural Stories self-paced reading data and **(B)** Dundee eye-tracking data. The difference in by-item squared error between the *GPT2S* and *GPT2L* models is significant at *p* < 0.05 level for the self-paced reading data, and the difference in by-item squared error between the *GPT2S* and *GPT2M* models is significant at *p* < 0.05 level for the eye-tracking data.

The CDR results from the different variants of the GPT-2 model in Figure 6 replicate the results from LME regression and show a positive correlation between test perplexity and predictive power on both self-paced reading times and eye-gaze durations. This provides further support for the observation that the trend in which neural LMs with lower perplexity predict latency-based measures more accurately may be mostly driven by the difference in their primary architecture or the amount of data used for training. The replication of these results may also suggest that neural LMs with higher model capacity are able to make accurate predictions about the upcoming word while relying less on humanlike generalizations given the same amount of training data.

## 8. Experiment 4: Effect of Predictability Over Word Frequency

In all previous experiments, only predictors that capture low-level cognitive processing were included in the baseline regression models. Although this procedure allowed a clean comparison of the predictive power of surprisal estimates from different models, this did not shed light on whether or not they contribute a separable effect from word frequency, which has long been noted to influence processing difficulty (Inhoff and Rayner, 1986). The goal of this experiment is to evaluate the contribution of surprisal estimates on top of a stronger baseline regression model that includes word frequency as a predictor. To this end, the CDR analyses of the previous experiments were replicated with a stronger baseline model, following the same protocol of fitting baseline and full regression models and calculating the difference in their log-likelihoods (ΔLL).

### 8.1. Procedures

For self-paced reading times, eye-gaze durations, and BOLD signals, baseline CDR models were fitted to the held-out set using the baseline predictors described in Section 5.2.1, as well as unigram surprisal to incorporate word frequency. Unigram surprisal was calculated using the KenLM toolkit (Heafield et al., 2013) with parameters trained on the English Gigaword Corpus (Parker et al., 2009) and was scaled prior to regression modeling. Other baseline model specifications were kept identical to those of the previous experiments.

The full models include one surprisal predictor on top of this baseline model, which were calculated from the parsers and LMs (Section 5.2.2) as well as different variants of the pretrained GPT-2 models (Section 6.1). Similarly, the specifications of the full models were kept identical to those of the previous experiments. After all the regression models were fitted, ΔLL for each model was calculated by subtracting the log-likelihood of the baseline model from that of a full regression model that contains its surprisal estimates.

### 8.2. Results

**Figure 7:**
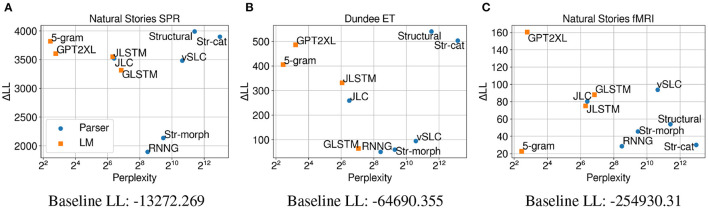
Perplexity measures from each model, and improvements in CDR model log-likelihood from including each surprisal estimate on **(A)** Natural Stories self-paced reading data, **(B)** Dundee eye-tracking data, and **(C)** Natural Stories fMRI data. The difference in by-item squared error between the *Structural* and *GPT2XL* models is significant at *p* < 0.05 level for the self-paced reading and fMRI data.

Figures 7A,B show that for self-paced reading times and eye-gaze durations, the ΔLL measures for most models indicate a substantial contribution of model surprisal on top of unigram surprisal. These results are consistent with Shain (2019), who observed that the effect of predictability subsumes that of word frequency in the context of naturalistic reading. The contribution of surprisal estimates are more subdued on fMRI data (Figure 7C), especially for the *5-gram* and *RNNG* models as well as the ablated variants of the *Structural* model.

On self-paced reading times, the ΔLL measures from the models in Figure 7A generally show a similar trend to the CDR results in Figure 5A. One notable difference, however, is that the ΔLL measures for the *GPT2XL* and *Str-cat* models are more comparable with those of other models when unigram surprisal is included in the baseline. This may be due to the fact that both the *GPT2XL* and *Str-cat* models incorporate subword information into their surprisal estimates (through their subword-level prediction and character-based word generation model, respectively) and therefore capture information that is more orthogonal to word frequency. On both eye-tracking and fMRI data, the overall trend is very similar to the CDR results in Figures 5B, 3C.

**Figure 8:**
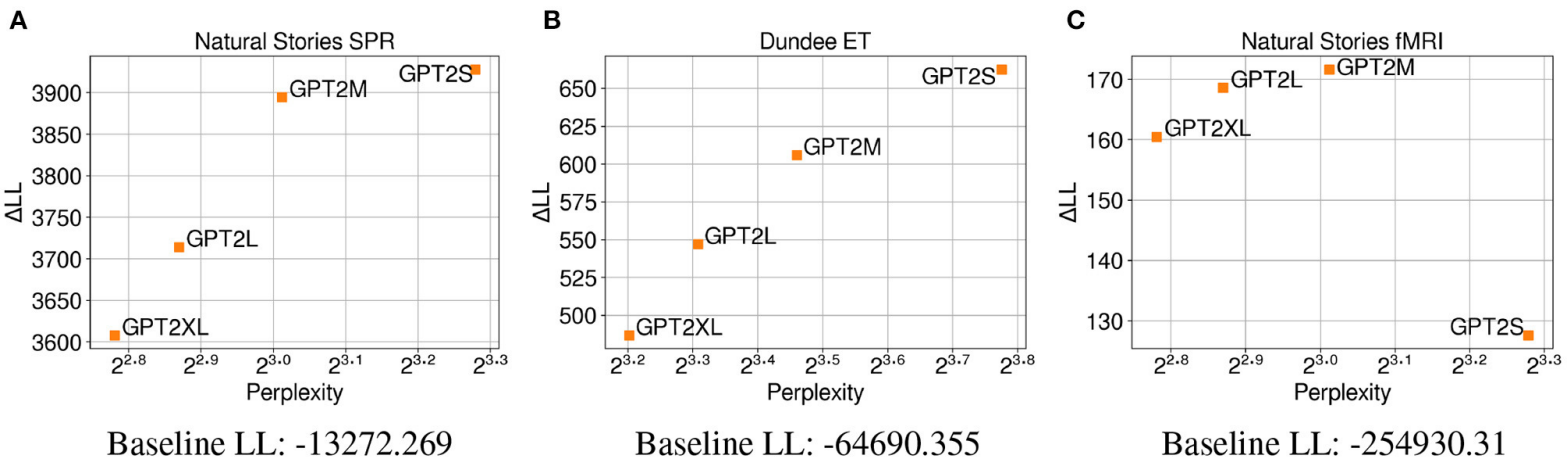
Perplexity measures from each GPT-2 model, and improvements in CDR model log-likelihood from including each surprisal estimate on **(A)** Natural Stories self-paced reading data, **(B)** Dundee eye-tracking data, and **(C)** Natural Stories fMRI data. The difference in by-item squared error between the *GPT2S* and *GPT2L* models is significant at *p* < 0.05 level for the self-paced reading data, and the difference in by-item squared error between the *GPT2S* and *GPT2M* models is significant at *p* < 0.05 level for the eye-tracking and fMRI data.

The CDR results from the different variants of the GPT-2 model in Figure 8 closely replicate the CDR results without unigram surprisal on all three datasets (Figures 4C, 6). This again shows a positive correlation between test perplexity and predictive power on self-paced reading times and eye-gaze durations. Additionally, this close replication across the three datasets shows that different model capacity does not result in surprisal estimates that are differentially sensitive to word frequency for the GPT-2 models.

## 9. Discussion and Conclusion

This article evaluates two kinds of NLP systems, namely incremental parsers and language models, as cognitive models of human sentence processing under the framework of expectation-based surprisal theory. As an attempt to develop a more cognitively plausible model of sentence processing, an incremental left-corner parser that explicitly incorporates information about common linguistic abstractions is first presented. The model is trained to make decisions about syntactic categories, predicate-argument structure, and morphological rules, which is expected to help it capture humanlike expectations for the word that is being processed.

The first experiment reveals that surprisal estimates from this structural model make the biggest contribution to regression model fit compared to those from other incremental parsers and LMs on self-paced reading times and eye-gaze durations. Considering that this model was trained on much less data in comparison to the LMs, this suggests that the strong linguistic generalizations made by the model help capture humanlike processing costs. This highlights the value of incorporating linguistic abstractions into cognitive models of sentence processing, which may not be explicit in LMs that are trained to predict the next word. Future work could investigate the contribution of discourse-level information in providing an explanation of humanlike processing costs (e.g., information about coreferential discourse entities; Jaffe et al., 2020). Additionally, perplexity measures from the evaluated models on the Natural Stories and Dundee corpora mostly support the negative monotonic relationship between LM perplexity and predictive power noticed in recent studies (Goodkind and Bicknell, 2018; Hao et al., 2020; Wilcox et al., 2020), although some incremental parsers deviate from this trend. The BOLD signals do not show a similar pattern to what was observed on latency-based measures, which indicates that they may be capturing different aspects of processing difficulty.

The second experiment compares the predictive power of surprisal estimates from different variants of GPT-2 models (Radford et al., 2019), which differ only by model capacity (i.e., number of layers and parameters) while holding the primary architecture (i.e., Transformers) and training data constant. The results show a robust *positive* correlation between perplexity and predictive power, which directly contradicts the findings of recent work. This indicates that the previously observed relationship between perplexity and predictive power may be driven more by the difference in the models' primary architecture or training data, rather than their capacity. Additionally, these results may suggest that when the training data is held constant, high-capacity LMs may be able to accurately predict the upcoming word while relying less on humanlike generalizations, unlike their lower-capacity counterparts.

The third experiment is a replication of the previous two experiments using continuous-time deconvolutional regression (CDR; Shain and Schuler, 2021), which is able to bring temporal diffusion under control by modeling the influence of a sequence of input predictors on the response. While there was no significant difference in the trend of predictive power among the different models for eye-gaze durations, the use of CDR made a notable difference in the results for self-paced reading times. This differential effect across datasets could be due to the fact that the regression models for eye-gaze durations include baseline predictors about the previous word sequence (i.e., “saccade length” and “previous word was fixated”). Additionally, the models that saw the biggest increase in ΔLL on self-paced reading times were LMs that are especially sensitive to the local context (i.e., *n*-gram models and LSTM models). Therefore, it can be conjectured that each by-word surprisal estimate from these models provides orthogonal information for the CDR model to make accurate predictions with. The positive correlation between perplexity and predictive power among the different variants of the GPT-2 model is still observed when CDR is used, providing further support for the robustness of this trend.

The final experiment is a replication of CDR analysis with a stronger baseline model, which included unigram surprisal as a predictor that reflects word frequency. For most models, the surprisal estimates contributed substantially to regression model fit on top of unigram surprisal, with their effects being stronger on self-paced reading times and eye-gaze durations. On self-paced reading times, the inclusion of unigram surprisal in the baseline resulted in more comparable ΔLL measures for the *GPT2XL* and *Str-cat* models, which hints at their capability to capture subword information. The general trend in ΔLL on eye-gaze durations and BOLD signals, as well as the positive correlation between perplexity and predictive power among the different variants of the GPT-2 model, was replicated.

Taken together, the above experiments seem to provide converging evidence that incremental parsers that embody strong generalizations about linguistic structure are more appropriate as computational-level models of human sentence processing. Although deep neural LMs have been shown to be successful at learning useful, domain-general language representations by the NLP community, they seem to require orders of magnitude more training data and yet do not provide a better fit to human reading behavior. In order for NLP to further inform cognitive modeling, future work should continue to focus on incorporating linguistic generalizations that are relevant into concrete models and evaluating their predictions on human subject data.

## Data Availability Statement

Publicly available datasets were analyzed in this study. This data can be found here: https://github.com/languageMIT/naturalstories (Natural Stories SPR), https://osf.io/eyp8q/ (Natural Stories fMRI).

## Author Contributions

B-DO: conceptualization, formal analysis, methodology, software, visualization, writing—original draft, and review and editing. CC: conceptualization, formal analysis, methodology, software, writing—original draft, and review and editing. WS: conceptualization, formal analysis, funding acquisition, methodology, project administration, resources, software, supervision, writing—original draft, and review and editing. All authors contributed to the article and approved the submitted version.

## Funding

This work was supported by the National Science Foundation Grant #1816891.

## Author Disclaimer

All views expressed are those of the authors and do not necessarily reflect the views of the National Science Foundation.

## Conflict of Interest

The authors declare that the research was conducted in the absence of any commercial or financial relationships that could be construed as a potential conflict of interest.

## Publisher's Note

All claims expressed in this article are solely those of the authors and do not necessarily represent those of their affiliated organizations, or those of the publisher, the editors and the reviewers. Any product that may be evaluated in this article, or claim that may be made by its manufacturer, is not guaranteed or endorsed by the publisher.

## Footnotes

^1^Johnson-Laird (1983) refers to lexical and grammatical decisions as “shift” and “predict”, respectively.

^2^The valence of a category is the number of unsatisfied syntactic arguments it has. Separate vectors for each syntactic argument are needed in order to correctly model cases such as passives where syntactic arguments do not align with predicate arguments.

^3^The predicates in this annotation scheme come from words that have been lemmatized by a set of rules that have been manually written and corrected in order to account for common irregular inflections.

^4^Participants of predications are numbered starting with zero so as to align loosely with syntactic arguments in canonical form.

^5^Only identity propagation is implemented in the experiments described in this article.

^6^The exploratory set contains data points whose summed subject and sentence number have modulo four equal to zero or one, and the held-out set contains data points whose summed subject and sentence number have modulo four equal to two or three.

^7^The partitioning for eye-gaze durations followed the same protocol as the self-paced reading times.

^8^For each participant, alternate 60-s intervals of BOLD series were assigned to the two partitions.

^9^The most likely sequence of parsing decisions from beam search decoding can also be used to construct parse trees. This model achieves a bracketing F1 score of 84.76 on WSJ22, 82.64 on WSJ23, 71.86 on Natural Stories, and 69.87 on Dundee. It should be noted that this performance is lower than the state-of-the-art partly because the model was trained on data with GCG-style annotation with hundreds of syntactic categories. For comparison, the parser from van Schijndel et al. (2013) achieves a bracketing F1 score of 85.20 on WSJ22, 84.08 on WSJ23, 69.60 on Natural Stories, and 70.66 on Dundee.

^10^ https://github.com/coryshain/cdr

^11^Perplexity of the parsers is higher partly because they optimize for a joint distribution over words and trees.

^12^This is analogous to the “index of fMRI sample” predictor for BOLD signals.

## Appendix

### 1. Predicate Context Composition Matrices

Predicate context vectors **h**_*a*_ and **h**_*b*_ for apex nodes *a* and base nodes *b* consist of 1 + *V* + *E* concatenated vectors of dimension *K*; one for the referential state signified by the sign itself, one for each of its *V* syntactic arguments, and one for each of its *E* non-local arguments (such as gap fillers or relative pronoun antecedents). Composition operators *o*_*g*_*t*__ may consist of zero or more unary operations (like extraction or argument reordering, which do not involve more than one child) followed by a binary operation. Composition matrices **A**_*o*_1_,*o*_2_, …_ and **B**_*o*_1_,*o*_2_, …_ with sequences of unary and binary operators *o*_1_, *o*_2_, … can be recursively decomposed:

$$\begin{array}{l} {\text{A}}_{o_{1},o_{2},\ldots }={\text{A}}_{o_{2},\ldots }\;{\text{U}}_{o_{1}}\\ {\text{B}}_{o_{1},o_{2},\ldots }={\text{B}}_{o_{2},\ldots }\;\left[\begin{array}{cc} {\text{U}}_{o_{1}} & 0^{H\times H}\\ 0^{H\times H} & {\text{I}}^{H\times H} \end{array}\right] \end{array}$$

where *H* = *K* + *KV* + *KE*. Each matrix is tiled together from identity matrices over predicate contexts that specify which syntactic or non-local arguments are associated between children (rows *u*) and parents (columns *v*).

Unary operators model extraction and argument swapping between a parent and a single child^13^:

$$\begin{array}{l} {\text{U}}_{\text{Ea-}n}=\overset{V+E}{\underset{u=0}{\sum }}\overset{V+E}{\underset{v=0}{\sum }}\delta_{u}\;\delta_{v}^{⊤}⊗\\ \;\{\begin{array}{ll} {\text{I}}^{K\times K} & \text{if }u=n\text{ and }v=V+1\\ {\text{I}}^{K\times K} & \text{if }u\neq n\text{ and }u\leq V\text{ and }v=u\\ {\text{I}}^{K\times K} & \text{if }u\neq n\text{ and }u>V\text{ and }v=u+1\\ 0^{K\times K} & \text{otherwise} \end{array} \end{array}$$

Left-child operator matrices model left arguments (Aa), left modifiers (Mb), gap filler attachments (G), left and right conjuncts (Ca, Cb), and other compositions:

$$\begin{array}{l} \;{\text{A}}_{\text{Aa-}n\text{-}e}=\overset{V+E}{\underset{u=0}{\sum }}\overset{V+E}{\underset{v=0}{\sum }}\delta_{u}\;\delta_{v}^{⊤}⊗\\ \;\{\begin{array}{ll} {\text{I}}^{K\times K} & \text{if }u=0\text{ and }v=n\\ {\text{I}}^{K\times K} & \text{if }u>V\text{ and }v=u\text{ and }e_{\left[v-V\right]}=0\\ 0^{K\times K} & \text{otherwise} \end{array}\\ \;{\text{A}}_{\text{Ma-}e}=\overset{V+E}{\underset{u=0}{\sum }}\overset{V+E}{\underset{v=0}{\sum }}\delta_{u}\;\delta_{v}^{⊤}⊗\\ \;\{\begin{array}{ll} {\text{I}}^{K\times K} & \text{if }u=1\text{ and }v=0\\ {\text{I}}^{K\times K} & \text{if }u>V\text{ and }v=u\text{ and }e_{\left[v-V\right]}=0\\ 0^{K\times K} & \text{otherwise} \end{array}\\ \;{\text{A}}_{\text{G}}=0^{H\times H}\\ {\text{A}}_{\text{Ca}},{\text{A}}_{\text{Cb}}={\text{I}}^{H\times H}\\ \;{\text{A}}_{o\text{-}e}=(\overset{V+E}{\underset{u=0}{\sum }}\overset{V+E}{\underset{v=0}{\sum }}\delta_{u}\;\delta_{v}^{⊤}⊗\\ \;\{\begin{array}{ll} {\text{I}}^{K\times K} & \text{if }u\leq V\text{ and }v=u\\ {\text{I}}^{K\times K} & \text{if }u>V\text{ and }v=u\text{ and }e_{\left[v-V\right]}=0\\ 0^{K\times K} & \text{otherwise} \end{array})\\ \text{ for all other }o\text{.} \end{array}$$

where *n* ∈ {1..*V*} is an argument number and *e* ∈ {0, 1}^*E*^ is a bit sequence encoding whether each non-local argument propagates to the left (0) or right (1) child.

Right-child operator matrices model right arguments (Ab), right modifiers (Mb), right relative clause attachments (Rb; introducing a non-local argument for a relative pronoun), left and right conjuncts (Ca, Cb), and other compositions:

$$\begin{array}{l} \;{\text{B}}_{\text{Ab-}n\text{-}e}=(\overset{V+E}{\underset{u=0}{\sum }}\overset{V+E}{\underset{v=0}{\sum }}\delta_{u}\;\delta_{v}^{⊤}⊗\\ \;\{\begin{array}{ll} {\text{I}}^{K\times K} & \text{if }u=0\text{ and }v=n\\ {\text{I}}^{K\times K} & \text{if }u>V\text{ and }v=u\text{ and }e_{\left[v-V\right]}=1\\ 0^{K\times K} & \text{otherwise} \end{array})\\ \;[{\text{I}}^{H\times H},{{\text{A}}_{\text{Ab-}n}}^{⊤}]\\ \;{\text{B}}_{\text{Mb-}e}=(\overset{V+E}{\underset{u=0}{\sum }}\overset{V+E}{\underset{v=0}{\sum }}\delta_{u}\;\delta_{v}^{⊤}⊗\\ \;\{\begin{array}{ll} {\text{I}}^{K\times K} & \text{if }u=1\text{ and }v=0\\ {\text{I}}^{K\times K} & \text{if }u>V\text{ and }v=u\text{ and }e_{\left[v-V\right]}=1\\ 0^{K\times K} & \text{otherwise} \end{array})\\ \;[{\text{I}}^{H\times H},{{\text{A}}_{\text{Mb}}}^{⊤}]\\ \;{\text{B}}_{\text{Rb}}=[0^{H\times H},\;(\overset{V+E}{\underset{u=0}{\sum }}\overset{V+E}{\underset{v=0}{\sum }}\delta_{u}\;\delta_{v}^{⊤}⊗\\ \;\{\begin{array}{ll} {\text{I}}^{K\times K} & \text{if }u=V+1\text{ and }v=1\\ {\text{I}}^{K\times K} & \text{if }u>V+1\text{ and }v=u-1\\ 0^{K\times K} & \text{otherwise} \end{array})]\\ {\text{B}}_{Ca},{\text{B}}_{Cb}=\left[{\text{I}}^{H\times H},{\text{I}}^{H\times H}\right]\\ \;{\text{B}}_{o\text{-}e}=(\overset{V+E}{\underset{u=0}{\sum }}\overset{V+E}{\underset{v=0}{\sum }}\delta_{u}\;\delta_{v}^{⊤}⊗\\ \;\{\begin{array}{ll} {\text{I}}^{K\times K} & \text{if }u\leq V\text{ and }v=u\\ {\text{I}}^{K\times K} & \text{if }u>V\text{ and }v=u\text{ and }e_{\left[v-V\right]}=1\\ 0^{K\times K} & \text{otherwise} \end{array})\\ \;\left[{\text{I}}^{H\times H},{{\text{A}}_{o\text{-}e}}^{⊤}\right]\text{ for other }o\text{.} \end{array}$$

where *n* ∈ {1..*V*} is an argument number and *e* ∈ {0, 1}^*E*^ is a bit sequence encoding whether each non-local argument propagates to the left (0) or right (1) child. Right-child matrices are of dimension *H* × 2*H* in order to accommodate associations between syntactic and non-local arguments in left children as well as parents.
